# Assessment of Knowledge, Attitudes, and Practices Regarding Microbiota Composition and Influencing Factors Among the Lebanese Population: A Cross-Sectional Study

**DOI:** 10.7759/cureus.109209

**Published:** 2026-05-19

**Authors:** Ahmad Moukachar, Ibrahim Khreis, Yara Tarhini, Antoine Abou Rached

**Affiliations:** 1 Faculty of Medical Sciences, Lebanese University, Beirut, LBN; 2 Internal Medicine and Gastroenterology, Faculty of Medical Sciences, Lebanese University, Beirut, LBN

**Keywords:** antibiotics, attitudes, knowledge, microbiota, practices, probiotics, public health

## Abstract

Background

The human microbiota, formed by bacteria, viruses, and yeasts, plays a fundamental role in maintaining health and preventing disease. It is influenced by multiple factors such as age, sex, hygiene practices, and dietary habits. Its disruption, particularly through inappropriate use of antibiotics or incorrect consumption of probiotics, may have important health consequences. Over the past two decades, growing scientific evidence has highlighted the essential role of microbiota in maintaining human health. Despite the growing interest in microbiota, public knowledge, attitudes, and practices regarding these concepts remain poorly understood, especially in Lebanon, where antibiotic self-medication and widespread availability of probiotic products are common. This study aimed to assess the knowledge of microbiota and probiotics, as well as attitudes and practices affecting the microbiota among Lebanese adults aged 18 to 65 years.

Methodology

A descriptive, cross-sectional study was conducted between July and December 2024 using a validated self-administered questionnaire, previously used in a similar study from Jazane, distributed online through a non-probability snowball sampling method. Lebanese adults aged 18 to 65 years were included. The questionnaire assessed sociodemographic characteristics, knowledge of microbiota and probiotics, and related attitudes and practices. Descriptive statistics were performed, categorical variables were reported as frequencies and percentages, while continuous variables were presented as means, medians, standard deviations, and ranges. Associations were analyzed using chi-square tests, Student’s t-test, or Mann-Whitney U test as appropriate. Pearson’s correlation analysis was used to assess the relationships between knowledge, attitudes, and practices. A p-value <0.05 was considered statistically significant.

Results

A total of 399 participants were included, with a mean age of 32.82, and 60.4% were females. Most participants resided in South Lebanon/Nabatiyeh (42.1%), and 88% held a university degree or higher. Good knowledge of microbiota was observed in 34.3% of participants, while only 17.3% demonstrated good knowledge of probiotics. Although 72.7% had heard of probiotics, misconceptions were frequent, particularly regarding their indications and safety. Good attitudes and practices were observed in 34.3% of participants. Notably, 34.8% reported using antibiotics without a prescription, and only 14.3% reported taking probiotics after completing an antibiotic course. Good microbiota knowledge was associated with good probiotics knowledge and positive attitudes and practices (p < 0.01).

Conclusions

While awareness of microbiota and probiotics is relatively high among the Lebanese population, true understanding remains limited. In addition, attitudes and practices were mixed, and important misconceptions and misuse persist. Knowledge gaps are reflected in suboptimal practices. Targeted educational interventions and public health strategies are needed to promote microbiota-friendly behaviors and improve long-term health outcomes.

## Introduction

The human body harbors trillions of microorganisms, collectively known as microbiota, which include bacteria, yeasts, and viruses. These microorganisms form a dynamic symbiotic relationship with host cells and colonize multiple body sites, such as the oral cavity, lungs, skin, and vagina, with the gastrointestinal (GI) tract representing the largest reservoir [1-3].

Over the past two decades, growing scientific evidence has highlighted the essential role of microbiota in maintaining human health. Beyond serving as a protective barrier against pathogens, the microbiota contributes to immune system modulation, nutrient metabolism, vitamin synthesis, regulation of gut barrier integrity, and even influences distant organs through complex axes such as the gut-brain and gut-lung axes [4].

The composition of the microbiota is highly individualized and varies throughout a person’s life. It is influenced by multiple factors such as age, sex, hygiene practices, and dietary habits. Alterations in the microbiota have been linked to a wide range of conditions, including cancer, cardiovascular and metabolic diseases, and various psychiatric disorders [2,5].

Antibiotics, while essential for treating bacterial infections, exert broad-spectrum effects that disturb both pathogenic and beneficial microorganisms, often leading to dysbiosis and long-term alterations in microbial composition [6,7]. A well-known consequence of antibiotic-induced dysbiosis is *Clostridioides difficile* infection, which can be severe and potentially life-threatening [8].

Conversely, growing interest has emerged in improving host health by modulating the microbiota through probiotics [9,10]. The International Scientific Association for Probiotics and Prebiotics (ISAPP) defines probiotics as “live microorganisms which, when administered in adequate amounts, confer a health benefit on the host” [11]. Initially proposed by Metchnikoff, probiotics have demonstrated multiple health benefits. These include competing with pathogens for adhesion sites and nutrients, enhancing mucosal integrity and gut barrier function, modulating gastrointestinal motility, and synthesizing antimicrobial compounds and bacteriocins [10]. As a result, they have been used in managing a variety of health conditions [12].

Given the essential role of microbiota in health and disease, any disruption due to environmental changes, lifestyle choices, or medications such as antibiotics and probiotics can have significant health consequences. This issue is increasingly concerning, considering the rising unsupervised and over-the-counter use of these agents. Therefore, it is imperative to promote comprehensive knowledge and appropriate practices related to the microbiota and its modulators.

However, despite the expanding body of scientific knowledge on microbiota and its modulators, public understanding of these concepts remains limited worldwide. For instance, a study conducted in the United Arab Emirates revealed only average knowledge and suboptimal attitudes and practices toward microbiota-related topics [2]. Similarly, a study in Saudi Arabia found average awareness of microbiota, poor understanding of probiotics, and largely neutral attitudes toward both probiotics and antibiotics [13].

In Lebanon, where over-the-counter antibiotic use and self-medication practices remain common, and where probiotic products are widely available [14,15], to our knowledge, no previous study has evaluated the population’s knowledge, attitudes, and practices regarding microbiota and its influencing factors. Assessing these parameters is essential for identifying misconceptions, unsafe behaviors, and knowledge gaps that may contribute to dysbiosis-related health risks. Such information is crucial for designing targeted educational interventions and public health strategies aimed at promoting appropriate antibiotic use, informed probiotic consumption, and overall microbiota-friendly practices.

Therefore, this study aimed to assess the knowledge of microbiota and probiotics, as well as attitudes and practices affecting the microbiota among Lebanese adults aged 18 to 65 years. Additionally, the study sought to evaluate the associations between these outcomes and selected sociodemographic characteristics.

## Materials and methods

Study design

A descriptive, cross-sectional study was conducted in Lebanon from July to December 2024 to evaluate the knowledge, attitudes, and practices of the Lebanese population concerning the human microbiota and its influencing factors, particularly probiotics and antibiotics.

Study population

The study targeted Lebanese citizens residing in Lebanon, aged between 18 and 65 years, and capable of providing informed consent. Individuals who declined participation or did not meet the eligibility criteria were excluded. Recruitment was conducted across all Lebanese governorates using a non-probability snowball sampling method.

The minimum required sample size was calculated using the Centers for Disease Control and Prevention Epi Info version 7.2.6 for population surveys. Assuming an expected frequency of 50%, a 95% confidence level, and a 5% margin of error, the required sample size was estimated at 384 participants. A total of 404 responses were received, of which 399 were eligible for final analysis.

Data collection

In this cross-sectional study, a structured approach was implemented to recruit participants, ensuring informed consent before questionnaire completion. Participants were provided with a brief explanation of the study, followed by a consent confirmation question. Data were collected via an online Google Forms questionnaire distributed through WhatsApp and various social media platforms. Furthermore, participants were encouraged to share the survey with others using the snowball technique, thus aiming to reach people from all Lebanese districts.

A validated, self-administered questionnaire, available in both Arabic and English, was adopted from a previous study conducted in the Jazan province in 2023 [13]. Its internal consistency was reported with a Cronbach’s alpha of 0.788, indicating acceptable reliability.

The final survey consisted of 42 questions, divided into four sections. First, sociodemographic characteristics were collected, including age, gender, place of residence, education level, employment status, and crowding index. Knowledge of the microbiota was assessed using 12 items, including three general closed-ended questions that were not scored, and nine true/false/don’t know questions, score-based questions, that were used to assess specific knowledge regarding microbiota definition, composition, and functions. One point was awarded for each correct answer, and zero points for each incorrect/don’t know answer. Total scores were categorized as poor (<3), average (3-6), or good (7-9). In addition, knowledge of probiotics was evaluated using 14 items, five general closed-ended questions, and nine scored true/false/don’t know questions, with the same scoring method mentioned above. Finally, attitudes and practices were measured via nine items using a three-point Likert scale, with a score of 1 assigned to “Unlikely,” 2 to “Neutral,” and 3 to “Likely” for positive practices, and conversely, a score of 3 for “Unlikely,” 2 for “Neutral,” and 1 for “Likely” for negative practices. Scores were interpreted as follows: bad (9-15), neutral (16-21), and good (22-27).

Ethical considerations

Ethical approval was obtained from the Institutional Review Board (IRB) of the Lebanese Hospital Geitaoui-University Medical Center (LHG-UMC) before the initiation of the study (IRB code: 2024-IRB-018). Participation was entirely voluntary. Electronic informed consent was obtained before proceeding with the questionnaire. Participant anonymity, privacy, and confidentiality were rigorously maintained; no identifiable data were collected, and access to the dataset was restricted to the research team.

Data analysis

Of the 404 responses received, 399 were retained for final analysis. Three participants were excluded because they did not meet the age criteria, and two were removed due to implausible responses. The data collected were entered and coded into Microsoft Excel (Microsoft Corp., Redmond, WA, USA) and subsequently analyzed using SPSS statistical software version 25 (IBM Corp., Armonk, NY, USA). Descriptive statistics were conducted where categorical variables were reported as frequencies and percentages, while continuous variables were presented as means, medians, standard deviations, and ranges. Moreover, for categorical variables, the chi-square tests were employed to assess associations between sociodemographic variables and knowledge levels of microbiota and probiotics, whereas the analysis of variance test was used with continuous variables. Additionally, Pearson’s chi-square test was used to evaluate the associations between knowledge, attitudes, and practices. A p-value <0.05 was considered statistically significant.

## Results

Sociodemographic characteristics

A total of 399 participants were included in the analysis. Table 1 summarizes their sociodemographic characteristics. The mean age of participants was 32.82 years (SD = 12.55), with a predominance of females (n = 241, 60.4%). The majority resided in South Lebanon/Nabatiyeh (168 (42.1%)), followed by Beirut (103 (25.8%)). Regarding occupational status, 148 (37.1%) were unemployed, 173 (43.4%) were employed in non-healthcare fields, and 78 (19.5%) were healthcare professionals. Most participants held a university degree or higher (351 (88%)). The mean crowding index was 1.03 (SD = 0.43).

**Table 1:** Sociodemographic characteristics of the study population. N: number; SD: standard deviation

Characteristics		N (%) or mean (SD)
Gender, N (%)	Female	241 (60.4)
	Male	158 (39.6)
Age, mean (SD)		32.82 (12.55)
Governorate, N (%)	Beirut	103 (25.8)
	Bekaa/Baalbek-Hermel	20 (5)
	Mount Lebanon	95 (23.8)
	North Lebanon/Akkar	13 (3.3)
	South Lebanon/Nabatiyeh	168 (42.1)
Occupation, N (%)	Unemployed	148 (37.1)
	Non-healthcare worker	173 (43.4)
	Healthcare worker	78 (19.5)
Educational level, N (%)	High school or below	48 (12)
	University graduate	169 (42.4)
	Postgraduate degree	119 (29.8)
	Doctor of general medicine, dentistry, or pharmacy	63 (15.8)
Crowding index, mean (SD)		1.03 (0.43)

Knowledge of microbiota

As shown in Table 2, the mean knowledge score regarding microbiota was 5.26 (SD = 2.22). Although most participants (350 (87.7%)) correctly identified the term microbiota, only 137 (34.3%) participants demonstrated good knowledge, 203 (50.9%) average, and 59 (14.8%) poor knowledge.

**Table 2:** Participants’ knowledge, attitudes, and practices regarding microbiota and probiotics. N: number; SD: standard deviation

		N (%) or mean (SD)
Knowledge of microbiota	Total scores, mean (SD)	5.26 (2.22)
	Poor knowledge, N (%)	59 (14.8)
	Average knowledge, N (%)	203 (50.9)
	Good knowledge, N (%)	137 (34.3)
Knowledge of probiotics	Total scores, mean (SD)	4.11 (2.46)
	Poor knowledge, N (%)	100 (25.1)
	Average knowledge, N (%)	230 (57.6)
	Good knowledge, N (%)	69 (17.3)
Attitudes and practices toward microbiota	Total scores, mean (SD)	19.7 (3.49)
	Bad attitude, N (%)	41 (10.3)
	Neutral attitude, N (%)	221 (55.4)
	Good attitude, N (%)	137 (34.3)

Most participants correctly identified that bacteria are normally present on the skin (n = 367, 92%), and 291 (72.9%) knew that microbiota can protect against disease. However, misconceptions were evident. As shown in Table 3, only 201 (50.4%) participants correctly recognized that intestinal bacteria do not necessarily cause diarrhea, and only 79 (19.8%) correctly identified that oral bacteria do not inevitably cause bad breath.

**Table 3:** Participant responses to statements on microbiota and probiotics. N: number

Statements	Correctly, N (%)	Incorrectly, N (%)
It is normal to have bacteria on our skin (True)	367 (92%)	32 (8%)
Having bacteria in our nose is dangerous (False)	253 (63.4%)	146 (36.6)
Microbiota can protect against disease (True)	291 (72.9%)	108 (27.1)
Having bacteria in the intestine will cause diarrhea (False)	201 (50.4%)	198 (49.6%)
Microbiota improves one’s mood and state of mind (True)	115 (28.8%)	284 (71.2%)
It is good to have bacteria in our intestines (True)	282 (70.7%)	117 (29.3%)
Microbiota is mainly composed of chemicals (False)	241 (60.4%)	158 (39.6%)
Microbiota can cause disease (True)	272 (68.2%)	127 (31.8%)
Having bacteria in the mouth will cause bad breath (False)	79 (19.8%)	320 (80.2%)
Antibiotics are made from probiotics (False)	155 (38.8%)	244 (61.2%)
Probiotics help restore the balance of “good” and “bad” bacteria in the gut (True)	287 (71.9%)	112 (28.1%)
Probiotics cause constipation and diarrhea (False)	186 (46.6%)	213 (53.4%)
Some foods (yogurt and honey) can be sources of probiotics (True)	291 (72.9%)	108 (27.1%)
Probiotics are used to treat insomnia (False)	66 (16.5)	333 (83.5%)
Probiotics can protect against diseases such as heart disease, diabetes, and cancer (True)	156 (39.1%)	243 (60.9%)
Probiotics can never cause disease in humans (False)	136 (34.1%)	263 (65.9%)
Probiotics boost the immune system (True)	256 (64.2%)	143 (35.8%)
Probiotics and antibiotics can never be used together (False)	110 (27.6%)	289 (72.4%)

Furthermore, Table 4 shows that age was significantly associated with knowledge level (p = 0.005). Participants with good knowledge were younger on average (30.01 years) compared to those with poor (34.03 years) or average (34.3 years) knowledge. Governorate of residence was also significant (p = 0.022), with the highest proportion of good knowledge in North Lebanon/Akkar (n = 9, 69.2%). In addition, both occupation and education demonstrated a strong association (p < 0.001). Among healthcare workers, 57 (73.1%) had good knowledge. Similarly, 51 (81%) doctors of general medicine, dentistry, or pharmacy demonstrated good knowledge. In contrast, neither gender (p = 0.169) nor the crowding index (p = 0.556) showed a significant association with microbiota knowledge.

**Table 4:** Sociodemographic factors associated with knowledge of microbiota. The chi-square test was used to determine the associations between categorical variables and knowledge of microbiota, with χ² reported as the test value. Analysis of variance was used to determine the associations between continuous variables and knowledge of microbiota, with F reported as the test value. N: number; SD: standard deviation

Characteristics		Knowledge of microbiota			P-value	Test value
		Poor knowledge	Average knowledge	Good knowledge		
Gender, N (%)	Female	29 (12)	127 (52.7)	85 (35.3)	0.159	3.762
	Male	30 (19)	76 (48.1)	52 (32.9)		
Age, mean (SD)		34.03 (14.9)	34.34 (12.06)	30.01 (11.7)	0.005	5.304
Governorate, N (%)	Beirut	17 (16.5)	52 (50.5)	34 (33)	0.022	17.849
	Bekaa/Baalbek-Hermel	1 (5)	11 (55)	8 (40)		
	Mount Lebanon	9 (9.5)	45 (47.4)	41 (43.2)		
	North Lebanon/Akkar	1 (7.7)	3 (23.1)	9 (69.2)		
	South Lebanon/Nabatiyeh	31 (18.5)	92 (54.8)	45 (26.8)		
Occupation, N (%)	Unemployed	33 (22.3)	71 (48)	44 (29.7)	<0.001	76.458
	Non-healthcare worker	26 (15)	111 (64.2)	36 (20.8)		
	Healthcare worker	0	21 (26.9)	57 (73.1)		
Education level, N (%)	High school or below	14 (29.1)	24 (50)	10 (20.8)	<0.001	87.121
	University graduate	28 (16.6)	99 (58.6)	42 (24.9)		
	Postgraduate degree	17 (14.2)	68 (57.1))	34 (28.5)		
	Doctor of general medicine, dentistry, or pharmacy	0	12 (19)	51 (81)		
Crowding index, mean (SD)		0.99 (0.33)	1.02 (0.49)	1.06 (0.37)	0.556	0.587

Knowledge and use of probiotics

Although 290 (72.7%) participants had heard of probiotics, Table 2 shows that only 69 (17.3%) demonstrated good knowledge, with a mean score of 4.11 ± 2.46. The most common source of information about probiotics was healthcare providers (physicians, pharmacists, or nutritionists) (n = 127 (31.8%) participants). More than half (223 (55.9%)) correctly identified probiotics as microorganisms, and 291 (72.9%) recognized yogurt and honey as probiotic sources. However, important misconceptions were noted: 333 (83.5%) participants incorrectly believed probiotics could treat insomnia, and 289 (72.4%) believed probiotics and antibiotics should not be used together, as shown in Table 3.

**Figure 1:**
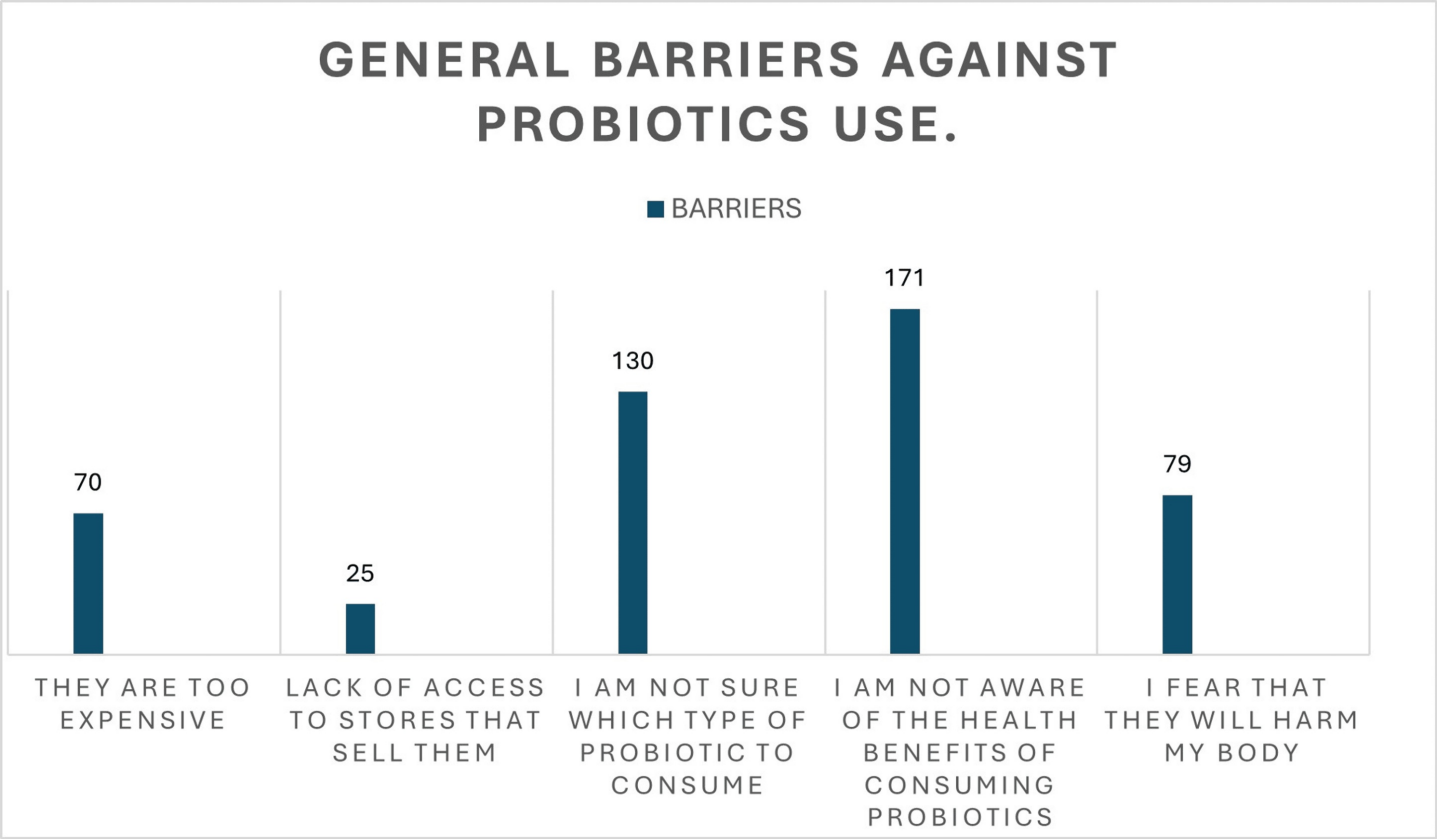
Reported barriers to probiotic use among participants.

Regarding usage, only 74 (18.5%) had previously used probiotics. Among non-users, 120 (30.1%) expressed no interest in trying them, whereas 205 (51.4%) were open to it. Figure 1 shows that the main barrier to probiotic use was a lack of awareness of their health benefits (n = 171, 42.8%), followed by the uncertainty about which type to consume (n = 130, 32.5%), the fear of potential harm (n = 79, 19.7%), their cost (n = 70, 17.5%), and the lack of access to stores that sell them (n = 25, 6.2%).

Table 5 represents the sociodemographic characteristics associated with probiotics knowledge. A higher proportion of males (50 (31.6%)) had poor knowledge than females (50 (20.7%)) (p = 0.018). Furthermore, participants with good knowledge were younger on average (29.5 years) than those with average (32.8 years) or poor (34.9 years) knowledge (p = 0.024). Geographic differences also played a role (p = 0.006); the highest rates of good knowledge were observed in Beirut (23 participants, 22.3%) and Mount Lebanon (19 participants, 20%), whereas Bekaa/Baalbek-Hermel and North Lebanon/Akkar had lower rates (n = 2 (10%) and 20 (11.9%), respectively). By occupation, 29 (37.2%) healthcare workers had good knowledge, and only 5 (6.4%) had poor knowledge, compared with much lower good knowledge rates among non-healthcare workers (16 (9.2%)) and 24 (16.2%) unemployed participants (p < 0.001). Similarly, education was another factor (p < 0.001); only 3 (6.2%) participants with a high school education or less showed good knowledge, compared to 24 (38.1%) with medical or dental doctorates. In contrast, the crowding index showed no significant association with probiotic knowledge (p = 0.954).

**Table 5:** Sociodemographic factors associated with knowledge of probiotics. The chi-square test was used to determine the associations between categorical variables and knowledge of probiotics, with χ² reported as the test value. Analysis of variance was used to determine the associations between continuous variables and knowledge of probiotics, with F reported as the test value. N: number; SD: standard deviation

Characteristics		Knowledge of probiotics			P-value	Test value
		Poor knowledge	Average knowledge	Good knowledge		
Gender, N (%)	Female	50 (20.7)	152 (63.1)	39 (16.2)	0.018	8.066
	Male	50 (31.6)	79 (49.4)	30 (19)		
Age, mean (SD)		34.9 (12.8)	32.8 (12.8)	29.5 (10.48)	0.024	3.748
Governorate, N (%)	Beirut	21 (20.4)	59 (57.3)	23 (22.3)	0.006	21.496
	Bekaa/Baalbek-Hermel	4 (20)	14 (70)	2 (10)		
	Mount Lebanon	16 (16.8)	60 (63.2)	19 (20)		
	North Lebanon/Akkar	1 (7.7)	7 (53.8)	5 (38.5)		
	South Lebanon/Nabatiyeh	58 (34.5)	90 (53.6)	20 (11.9)		
Occupation, N (%)	Unemployed	40 (27)	84 (56.8)	24 (16.2)	<0.001	38.685
	Non-healthcare worker	55 (31.8)	102 (59)	16 (9.2)		
	Healthcare worker	5 (6.4)	44 (56.4)	29 (37.2)		
Education level, N (%)	High school or below	22 (45.8)	23 (47.9)	3 (6.2)	<0.001	50.323
	University graduate	40 (23.7)	103 (60.9)	26 (15.4)		
	Postgraduate degree	37 (31.1)	66 (55.4)	16 (13.4)		
	Doctor of general medicine, dentistry, or pharmacy	1 (1.6)	38 (60.3)	24 (38.1)		
Crowding index, mean (SD)		1.02 (0.42)	1.03 (0.46)	1.04 (0.33)	0.954	0.048

Attitudes and practices toward microbiota

Table 2 summarizes participants’ attitudes and practices toward microbiota. The mean attitude/practice score was 19.7 (SD = 3.49). Good attitudes were observed in 137 (34.3%) participants, neutral attitudes in 221 (55.4%), and poor attitudes in 41 (10.3%).

Regarding antibiotic practices, Table 6 shows that 139 (34.8%) participants reported using antibiotics without a prescription, 94 (23.6%) reported stopping antibiotics once they felt better, and 70 (17.5%) reported taking antibiotics as a preventive measure. In contrast, 182 (45.6%) reported taking yogurt or probiotics after diarrhea or vomiting, but only 57 (14.3%) reported taking probiotics after completing an antibiotic course. Furthermore, good attitudes were observed in 93 (38.6%) females compared with 44 (27.8%) males (p = 0.004). Occupation and education were also strongly associated (p < 0.001). Of healthcare professionals, 41 (52.6%) had good attitudes, compared with 52 (35.1%) unemployed individuals and only 44 (25.4%) non-healthcare workers. In addition, good attitudes were most frequent among doctoral participants (n = 46, 57.1%) and least frequent among those with a high school education or less, with only 8 (17%) participants. No significant associations were observed with age (p = 0.135), governorate of residence (p = 0.299), or crowding index (p = 0.39).

**Table 6:** Attitudes and practices related to microbiota. N: number

Statement, N (%)	Likely	Neutral	Unlikely
I use antibiotics without a prescription	139 (34.8%)	48 (12%)	212 (53.1%)
I recommend antibiotics to my friends and family	74 (18.5%)	107 (26.8%)	218 (54.6%)
I stop taking antibiotics when I feel better, even if I am supposed to take them for a while longer	94 (23.6%)	49 (12.3%)	256 (64.2%)
I would take antibiotics as a precaution, so I do not get sick	70 (17.5%)	54 (13.5%)	275 (68.9%)
I take yoghurt or probiotics pills after having diarrhea or vomiting	182 (45.6%)	105 (26.3%)	112 (28.1%)
I would give my child probiotics	110 (27.6%)	150 (37.6%)	139 (34.8%)
I would give probiotics to an elderly family member	143 (35.8%)	143 (35.8%)	113 (28.3%)
I take probiotics pills or yogurt to improve my health	207 (51.9%)	103 (25.8%)	89 (22.3%)
After completing my antibiotics course, I would take probiotic pills	57 (14.3%)	223 (55.9%)	119 (29.8%)

Association between knowledge of microbiota and knowledge, attitudes, and practices regarding probiotics

As shown in Table 7, a statistically significant positive association was observed between microbiota and both probiotic knowledge and attitudes/practices related to microbiota (p < 0.001). Among participants with poor microbiota knowledge, 26 (44.1%) also had poor probiotic knowledge, whereas only 2 (3.4%) demonstrated good knowledge. Conversely, 48 (35%) of those with good microbiota knowledge also showed good probiotic knowledge, and only 17 (12.4%) scored poorly. Similarly, 68 (49.6%) participants with good microbiota knowledge also had good attitudes, while 60 (43.8%) had neutral attitudes, and only 9 (6.6%) had poor attitudes.

**Table 7:** Associations between knowledge of microbiota and probiotic knowledge, attitudes, and practices. The chi-square test was used to determine the associations between knowledge of microbiota and both probiotics knowledge and related attitudes and practices. N: number

		Knowledge of microbiota			P-value	χ²
		Poor knowledge, N (%)	Average knowledge, N (%)	Good knowledge, N (%)		
Knowledge of probiotics	Poor knowledge	26 (44.1)	57 (28.1)	17 (12.4)	<0.001	58.651
	Average knowledge	31 (52.5)	127 (62.6)	72 (52.6)		
	Good knowledge	2 (3.4)	19 (9.4)	48 (35)		
Attitudes and practices toward microbiota	Bad attitude	10 (16.9)	22 (10.8)	9 (6.6)	<0.001	25.168
	Neutral attitude	38 (64.4%)	123 (60.6)	60 (43.8)		
	Good attitude	11 (18.6)	58 (28.6)	68 (49.6)		

## Discussion

This study is the first from Lebanon to assess the knowledge, attitudes, and practices of the general population regarding microbiota and its major modulators. The study included 399 participants whose mean age was 32.82 years, with 241 (60.4%) females. The majority were employed in non-healthcare fields (173, 43.4%), and 78 (19.5%) were healthcare professionals. Most participants held university degrees (169, 42.4%), reflecting a relatively educated sample.

Regarding the knowledge of microbiota and probiotics, almost half of our participants demonstrated average knowledge (n = 203, 50.9%, and n = 230, 57.6%, respectively). People who had poor knowledge of probiotics were the least likely to have good knowledge of microbiota (2, 3.4%). Attitudes and practices were mostly neutral (221, 55.4%). Additionally, people with good attitudes and practices tended to have the best knowledge on microbiota (68, 49.6%).

While 350 (87.7%) participants correctly identified the term “microbiota,” 203 (50.9%) had average knowledge, 137 (34.3%) had good knowledge, and the minority (59 (14.8%)) had poor knowledge. This suggests that correct recognition of terminology does not necessarily translate into comprehensive understanding. Our results come in contrast with those of Rajab et al., who reported that, despite 898 (79.8%) participants correctly defining microbiota, only 165 (14.7%) participants showed good knowledge, and, similar to our findings, half had average knowledge [13]. In addition, our findings exceeded those of Barqawi et al., where 94 (29.3%) had good microbiota knowledge [2].

Participants generally demonstrated basic knowledge of certain fundamental aspects of microbiota, such as its normal presence on the skin (n = 367, 92%) and its commensal role in the intestines (n = 282, 70.7%). However, misconceptions were frequent: 198 (49.6%) believed microbiota causes diarrhea, and 320 (80.2%) associated microbiota with halitosis. Similar misconceptions were reported by Barqawi et al. and Rajab et al., in which many respondents believed that nasal bacteria are dangerous and that intestinal bacteria cause diarrhea [2,13]. Nonetheless, most participants understood that microbiota can simultaneously cause and protect against disease (n = 272 (68.2%) and n = 291 (72.9%), respectively). This indicates a decent level of awareness toward the role of microbiota and their potential pathogenicity in some cases, with room for improvement and correction of certain misconceptions and beliefs. Furthermore, younger participants were more likely to demonstrate good knowledge. However, age groups were very close in range, which limits the strength and interpretability of this result.

Healthcare workers had the highest percentage of good knowledge (n = 57, 73.1%) compared to non-healthcare workers and unemployed individuals. These findings are inconsistent with those of Barqawi et al., who reported that almost half of healthcare professionals had average knowledge [2]. Interestingly, according to the education level, among doctors of general medicine, dentistry, or pharmacy, 51 (81%) had good knowledge compared to only 19% with average knowledge, which is expected given their formal training in microbiology, infectious diseases, and related biomedical sciences, as well as their clinical exposure.

In contrast, only 42 (24.9%) of university graduates had good knowledge, suggesting a substantial gap outside health-related fields. This likely reflects limited exposure to microbiota-related concepts in non-medical education and reliance on non-structured sources of information. Overall, the results highlight that microbiota knowledge is strongly associated with health-related education and point to the need for broader awareness initiatives targeting non-medical graduates.

As the two are strongly interconnected, knowledge of microbiota was also affected by the level of knowledge of probiotics. Of the people who displayed good knowledge of microbiota, 72 (52.6%) showed average knowledge of probiotics, and 48 (35%) had good knowledge. This makes sense as the importance of probiotics lies in their role in affecting microbiota composition, reflecting the conceptual interconnection between the two domains.

Our findings suggest that the population has a basic understanding of microbiota, but misconceptions cannot be overlooked, and addressing them can help increase knowledge on this subject. It is also crucial to encourage doctors in the healthcare field to deepen their literature on the topic so they can implement better practices and contribute to the public’s education.

Regarding probiotics, only 69 (17.3%) participants demonstrated good knowledge. This result is comparable to findings by Al-Fawares et al., where 36 (13.3%) participants showed good knowledge [16], but is higher than those reported by Rajab et al., where only 28 (2.5%) had good knowledge. In addition, our study showed that only 100 (25.1%) responders had poor knowledge, a much lower percentage than that reported by Rajab et al., where the majority of responders (n = 860, 76.4%) had poor knowledge [13]. The majority of our participants had heard of the term “probiotics,” unlike those in a study conducted in Al-Qassim, where 495 (74%) responders denied familiarity with the term [17]. Additionally, 223 (55.5%) correctly defined probiotics as microorganisms (bacteria), a similar result was demonstrated by Rajab et al [13]. Despite this recognition, fewer than 20% had good knowledge of them, which might reflect superficial awareness and a lack of deep understanding of these beneficial microorganisms.

Our findings regarding knowledge about probiotics suggest that while the general population shows positive results regarding some aspects of probiotics, it still lacks in others. Participants generally recognized certain benefits of probiotics, such as boosting the immune system and balancing gut bacteria. Similarly, a Chinese study showed that the majority of the population correctly recognized their advantages [18]. In addition, almost half of our participants (182 (45.6%)) reported taking yoghurt or probiotic pills after having diarrhea or vomiting, showing their recognition of probiotics’ role in these GI disturbances. However, misconceptions were identified; many believed that probiotics cannot be taken with antibiotics and that they can treat unrelated conditions such as insomnia. Furthermore, 263 (65.9%) believed probiotics can never cause disease, overlooking documented risks in immunocompromised individuals. This highlights the importance of clarifying the indications and benefits of probiotics while addressing their potential risks and side effects to promote responsible and safe usage.

Younger participants demonstrated a higher level of knowledge; comparable results were also seen by Rajab et al. [13]. This can be explained by their access and more frequent exposure to social media and healthcare education platforms compared to older participants. Due to their medical background, healthcare workers rose to expectations and exhibited the highest proportion of good knowledge, with 29 (37.2%) participants, compared to their counterparts. This knowledge gap was also evident in studies by Barqawi et al. [2] and Rajab et al. [13]. Conversely, a study in Pakistan found that only 61 (15.1%) healthcare professionals had good knowledge of probiotics [19].

In addition, it is generally expected that individuals with limited formal education exhibit less awareness or understanding of the subject compared to those with higher education. This is supported by the lowest percentage of good knowledge (n = 3, 6.2%) demonstrated by individuals with the lowest educational level, a high school degree or below. Doctors of general medicine, dentistry, or pharmacy demonstrated the highest percentages (n = 24, 38.1%) of good knowledge compared to others. In addition, 129 (76.3%) university graduates had average knowledge or higher. Comparable results were found in India, where the average score on probiotic knowledge among college students was high [20], although the main source of information for the latter’s population was newspapers and TV, unlike our participants, whose knowledge was based mostly on healthcare providers, which is similar to the results reported by Ayyash et al. [21].

Regarding the population’s attitudes and practices, 221 (55.4%) had neutral attitudes, and 137 (34.3%) had good attitudes. These proportions were higher than those reported by Barqawi et al. and Rajab et al. [2,13]. Among our participants, we found that 205 (51.4%) responders were open to trying probiotics someday, and 12 (30.1%) had never tried them and were unwilling to do so, which is lower than those reported by Barqawi et al., with 185 (44.5%) unwilling to try them [2]. However, willingness to administer probiotics to vulnerable populations was limited: only 110 (27.6%) would give probiotics to their child, and 143 (35.8%) to an elderly family member. In contrast, Wang et al. reported that 1,778 (75.1%) parents believed probiotics positively affect children’s immunity [22]. This hesitation in practice, especially seen in these vulnerable groups, is mostly due to the lack of awareness about probiotics’ function and safety of use. This lack of awareness was also evident when asked about the barriers to using probiotics: the most common reason was their unawareness of the health benefits, followed by uncertainty about which probiotic to consume. The results corroborate those reported by Rajab et al. and Ayyash et al., where the most common barrier was also the lack of awareness of probiotics’ benefits [13,21]. Similarly, 38.63% of participants in a similar study from China were skeptical about the efficacy of probiotics, but their major barrier turned out to be the abundance of probiotics, making the consumer indecisive in choosing one [18]. From these results, we can conclude that awareness and education are key factors in implementing better attitudes and practices toward probiotics and, therefore, toward microbiota.

Concerning antibiotics, as stated in the literature, improper use of antibiotics is a leading cause of dysbiosis. Contrary to Barqawi et al., who did not find a single participant to show good attitudes or practices toward antibiotics [2], our population showed overall adequate attitudes and practices toward antibiotics. Almost half (212 (53.1%)) reported that they were unlikely to use antibiotics without a prescription, and 218 (54.6%) were unlikely to prescribe them to friends and family. This contrasts with Al-Shibani et al., who found that only 176 (8.9%) took them after a doctor’s prescription [23], but aligns with findings from Romania, where 572 (57.4%) consistently consulted their physician before using antibiotics [24].

Furthermore, 256 (64.2%) were unlikely to stop their antibiotic course before it is completed, despite symptomatic improvement, consistent with Mouhieddine et al., who found that 263 (53.1%) did not stop the course when their symptoms improved [14], but contradicting Al-Shibani et al., where 1,312 (67%) participants believed they should stop taking the antibiotic when they feel better [23].

These findings are more reassuring than studies from other countries where self-medication rates exceed 50%. Nevertheless, the persistence of unsafe behaviors highlights the need for continued public education and stricter regulation of antibiotic dispensing.

Among the sociodemographic factors, the occupation and educational level affected the attitude, aligning with findings from Mouheddine et al. [14]. This phenomenon seems reasonable as we assume that higher education means better knowledge, hence better attitudes.

Healthcare workers and doctors of general medicine, dentistry, or pharmacy were the categories that demonstrated the best attitude and practices. This study adds to previous studies that found that healthcare professionals tend to exhibit good attitudes. Some examples are Mouhieddine et al. and Abubakar et al., who found that almost half of the people who work in the healthcare sector showed a good attitude [14,25].

Finally, a positive association between microbiota knowledge and probiotics knowledge, attitudes, and practices. Participants with higher microbiota knowledge scores were more likely to demonstrate good probiotic knowledge and more favorable attitudes and practices toward probiotic use. These findings support the idea that knowledge serves as a foundational determinant influencing attitude and, subsequently, practices. This positive correlation underscores the importance of integrated educational interventions targeting both microbiota and probiotics, as improving conceptual understanding may translate into more responsible health behaviors.

The main strength of this study lies in the large sample size, meeting requirements for reliable statistical analysis and adequate statistical power. Second, the wide geographic coverage and representation, as it includes respondents from all Lebanese governorates. Furthermore, the use of a validated and tested questionnaire previously used in a similar study in a country enhances validity and reliability. Given the regional and cultural proximity between Saudi Arabia and Lebanon, as well as similarities in language (Arabic), educational background, and healthcare awareness levels within the target population, the instrument was considered appropriate for use in the Lebanese context without full re-validation. Finally, and most importantly, this study presented a novel contribution to the Lebanese public health literature and is one of the few in the Middle East, addressing an important gap in the Lebanese knowledge, as no prior assessment of microbiota knowledge and related behaviors has been published.

On the other hand, this study had some important limitations, one of which is its cross-sectional design, which prevents causality establishment. Another limitation is the use of online snowball sampling, which may have introduced selection bias and overrepresentation from certain regions, despite recruiting from all governorates, limiting the generalizability of the study. Third, the use of an online self-administered questionnaire, although necessary due to war-related and logistical constraints, may have introduced sampling bias, as individuals with internet access and higher educational backgrounds were more likely to participate in the study. The last two factors limit the generalizability of the study but were unavoidable due to the current state and logistical constraints imposed by the ongoing war in the country.

## Conclusions

In this study, most participants were familiar with the term “microbiota”; however, overall knowledge was mostly average, with notable misconceptions regarding its role and pathogenicity. In addition, a large proportion of participants were aware of probiotics, but only a minority demonstrated good knowledge. Regarding attitudes and practices, the findings demonstrated mixed practices, with both appropriate behaviors (such as consulting healthcare providers before antibiotic use and completing prescribed courses) and inappropriate practices (including self-medication, preventive use without medical indication, and early discontinuation of therapy) still present. It is important to note that the study sample was obtained through a non-probability online snowball sampling method and was relatively skewed toward highly educated individuals. Nevertheless, even within this relatively highly educated sample, important gaps in knowledge and suboptimal practices were still observed. Overall, these findings highlight the need for targeted educational interventions, public awareness campaigns, and strengthened involvement of healthcare professionals in promoting appropriate antibiotic use and improving microbiota-related health literacy. Integrating microbiota and probiotics education into academic curricula and public health strategies may further support long-term improvements in health behaviors.
